# Diagnostic Radiology Services and Occupational Radiation Anxiety in Kazakhstan

**DOI:** 10.3390/ijerph22121785

**Published:** 2025-11-25

**Authors:** Ainara Darbayeva, Tairkhan Dautov, Gulnur Zhakhina, Bakyt Duisenbayeva, Roza Suleimenova, Nurgali Nurmanbekov, Botagoz Nurseitova, Galiya Orazova

**Affiliations:** 1 Department of Epidemiology and Biostatistics, Astana Medical University, Astana 010000, Kazakhstan; aide26021986@gmail.com (A.D.);; 2 Radiology and Ultrasound Diagnostics Department, National Scientific Medical Center, Astana 010009, Kazakhstan; 3 Clinical and Academic Department of Radiology and Nuclear Medicine, University Medical Center, Astana 010000, Kazakhstan; 4 Department of Medicine, Nazarbayev University, Astana 010000, Kazakhstan; 5 Department Diagnostic Radiology, University Medical Center, Astana 010000, Kazakhstan; 6 Department of Inpatient Radiology, University Medical Center, Astana 010000, Kazakhstan; 7 Radiation Control Sector, University Medical Center, Astana 010000, Kazakhstan

**Keywords:** ionizing radiation, diagnostic imaging, radiology workforce, occupational exposure, anxiety

## Abstract

Radiology services play a vital role in modern healthcare, yet disparities in access and concerns about occupational radiation exposure remain understudied in many countries, including Kazakhstan. This study evaluates national trends in diagnostic imaging services, workforce distribution, and radiation-related anxiety among medical personnel. We analyzed national diagnostic imaging infrastructure and workforce data from 2018–2024. Individual radiation exposure data (*n* = 177) were obtained from dosimetry records in Astana’s medical facilities. Additionally, a cross-sectional survey (*n* = 324) was conducted using the Spielberger–Hanin Anxiety Scale to assess radiation-related anxiety and associated factors. Between 2018 and 2024, the number of CT rooms in Kazakhstan more than doubled from 162 to 358 (+121%), while X-ray examinations declined from 20.6 to 14.6 million (−29.2%) and fluorography dropped by 67.7%. CT scans increased over threefold, from 491,738 to 1.6 million. Radiologists grew from 3529 to 4511 (+27.8%), and ultrasound doctors from 1396 to 2178 (+56.1%). Interventional physicians had the highest quarterly radiation dose (0.65 ± 0.58 mSv, *p* = 0.001). Among radiology professionals, 32% reported anxiety related to occupational exposure. Anxiety was significantly associated with not using aprons (58% vs. 27%, *p* < 0.001), lack of dosimeter use (27% vs. 12%, *p* = 0.001), and inadequate safety training (27% vs. 6%, *p* < 0.001). Spielberger–Hanin scores ≥ 45 indicated high levels of situational (58%) and personal (56%) anxiety in this group. Kazakhstan’s diagnostic radiology capacity has grown rapidly, especially in CT availability, yet regional disparities and occupational anxiety remain critical concerns. Targeted workforce distribution, improved protective practices, and enhanced radiation safety education are urgently needed.

## 1. Introduction

Ionizing radiation, whether naturally occurring or human-made, is widely used in medicine and industry but carries significant health risks. Adverse effects on humans were identified soon after the discovery of X-rays, with early cases of non-cancerous tissue damage such as epilation, erythema, and skin burns reported in patients undergoing treatment for various conditions [1]. The carcinogenic effect of ionizing radiation exposure was proved by several epidemiological studies [2,3]. Beyond cancer, IR has been associated with non-lethal effects like cataracts and cardiovascular disease, observed in both animals and humans [4,5,6,7].

In medicine, ionizing radiation is an indispensable tool used in diagnostic imaging and therapeutic procedures. In the United States, medical imaging accounts for nearly half of the population’s total exposure to ionizing radiation [8]. Technologies such as X-rays, computed tomography (CT), fluoroscopy, and nuclear medicine significantly enhance clinical decision-making by enabling non-invasive visualization of internal structures. However, there may be health side effects. For example, computed tomography (CT) carries a small but measurable risk of cancer, which becomes a public health concern given the high volume of scans, especially among children who are more radiosensitive than adults [9]. Studies have linked pediatric CT exposure to increased risks of leukemia and brain tumors [10].

Medical personnel working in diagnostic radiology, particularly those involved in interventional procedures, are routinely exposed to low doses of ionizing radiation over prolonged periods. Ionizing radiation has been associated with a range of adverse health effects beyond cancer, including potential impacts on the nervous and cardiovascular systems. It may impair cognitive performance and compromise neurological function [11], and growing evidence links radiation exposure to a higher likelihood of developing dementia [12]. Recent research highlights that occupational radiation exposure could also elevate the risk of non-cancer conditions such as ischemic heart disease [13] and stroke [14,15], which are particularly relevant for medical professionals exposed over extended periods. In addition to these physical health risks, workers may experience psychological impacts, including heightened anxiety related to radiation exposure and uncertainty about long-term health consequences [16].

In Kazakhstan, where diagnostic imaging services have expanded significantly in recent years, national-level data on workforce distribution and diagnostic equipment availability are still limited, as are insights into occupational exposure and radiation safety practices. To address these gaps, this study presents a comprehensive national assessment of diagnostic radiology services and personnel, combined with a focused analysis of individual radiation exposure using dosimetry data from radiology departments in Astana, and a cross-sectional survey conducted among radiology professionals across the country.

## 2. Materials and Methods

### 2.1. Study Design

First, we present national-level data on the availability of diagnostic imaging facilities, radiology personnel, outpatient consultations, and imaging procedure volumes over recent years. We then assess the regional distribution of radiology physicians and imaging equipment per 100,000 population in 2024 to identify potential inequalities in access to diagnostic services. Next, we analyze characteristics and quarterly radiation doses of radiology personnel in the capital city, Astana (*n* = 177), by occupational category, highlighting differences in exposure across healthcare facility types (diagnostic and emergency healthcare facilities). Finally, we report findings from a survey of diagnostic radiology professionals addressing radiation risk awareness and anxiety levels (*n* = 324).

Kazakhstan employs radiation safety engineers who are responsible for collecting personal dosimeters from staff, submitting them to licensed laboratories for effective dose measurement, and receiving formal dose reports. Medical physicists are present in Kazakhstan; however, national data on their exact number were not available for this study.

By integrating system-level indicators with individual-level data, this research provides a comprehensive overview of diagnostic radiology services and radiation safety challenges in Kazakhstan. The findings are intended to inform national policy measures to optimize workforce planning, improve patient and staff safety, and ensure equitable access to high-quality imaging services.

### 2.2. Data Sources and Variables

This study utilizes comprehensive national-level data on diagnostic radiology services in Kazakhstan. The dataset includes annual aggregated statistics for the years 2018, 2020, 2022, and 2024. The data encompass various components of diagnostic radiology infrastructure and human resources, including the number of radiology rooms, computed tomography (CT) rooms, fluorography rooms, and fluorographic mobile units. Information is also provided on the number of radiology physicians (including specialists in X-ray diagnostics and CT), ultrasound (US) physicians, nuclear medicine physicians, and radiologic technologists (X-ray technicians).

All occupational dose measurements represented the effective dose, calculated by accredited external laboratories using thermoluminescent whole-body dosimeters worn on the chest. Dosimeters integrate cumulative exposure over three months. Because some personnel worked across more than four quarters in a year, dose values were analyzed per quarter to ensure comparability.

In addition, the dataset includes detailed information on service utilization, including the number of outpatient visits to radiologists, including cancer screening and disease-specific diagnostic consultations. To evaluate diagnostic imaging activity, the total annual number of procedures performed was analyzed. These include radiographic, fluorographic, CT, nuclear medicine (radioisotope) procedures, and ultrasound examinations.

All abovementioned data were obtained from the Salidat Kairbekova National Research Center for Health Development and aggregated at the national level to ensure comparability across years. The data on quarterly radiation doses for radiology personnel (*n* = 177) were collected from medical facilities in Astana, the capital city of Kazakhstan.

### 2.3. Survey Structure and Measurement Tools

A cross-sectional survey was conducted among 324 radiology department personnel across Kazakhstan (Supplementary File S1). The survey was conducted between 5–15 June 2025, by the snowball method. The questionnaire included demographic variables (age, gender, place of residence), occupational details (job type, work experience, weekly working hours), and variables related to radiation safety practices (use of dosimeters, protective equipment, prior radiation safety training).

Participants were informed they could withdraw from the survey at any time without explanation, and electronic informed consent was obtained from each participant.

The key independent variable was the response to the question “Are you worried or anxious about being exposed to ionizing radiation at work?” with three response options: “Yes,” “Sometimes,” and “No.” For analytical purposes, only “Yes” responses were categorized as indicating anxiety; both “Sometimes” and “No” were grouped together as “No anxiety.”

The psychological component of the survey employed Spielberger’s State-Trait Anxiety Inventory, a validated tool used to assess both state anxiety (situational, immediate anxiety) and trait anxiety (personal, general predisposition to anxiety) [17,18]. Each scale includes 20 items, and participants rate each item on a 4-point Likert scale. Total scores for each scale range from 20 to 80. Based on established thresholds, a score of 45 or higher is interpreted as high anxiety, while scores below 45 suggest low to moderate anxiety levels [18]. This tool has been widely used in occupational health research and is suitable for evaluating emotional responses to work-related stressors such as radiation exposure.

### 2.4. Statistical Analysis

Categorical variables were presented as frequencies and percentages for corresponding groups, while continuous variables were presented as means and standard deviations. To test the interaction between variables, Chi-squared test, Fisher’s exact, and Student’s *t*-tests were used where appropriate.

Availability of radiology specialists and inventory was presented per 100,000 population for each administrative division of Kazakhstan (17 regions and 3 cities). The corresponding maps were constructed for 2024 using QGIS 3.16.11 Hannover version.

All statistical analyses were performed using STATA 16.1 MP2 version (STATA Corporation, College Station, TX, USA). The study was approved by the Ethics Committee of NCJSC “Astana Medical University” (Protocol No. 3 from 28 May 2024). All survey respondents signed the consent form.

## 3. Results

### 3.1. Availability of Diagnostic Imaging Facilities

From 2018 to 2024, Kazakhstan has shown a consistent upward trend in the availability of diagnostic imaging infrastructure (Table 1). Radiology rooms increased from 1127 in 2018 to 1285 in 2024, representing a 14% increase. The most pronounced growth occurred in CT rooms, which more than doubled from 162 in 2018 to 358 in 2024 (+121%), reflecting a significant expansion in high-resolution imaging capabilities. Fluorography rooms also rose modestly from 815 to 877 (+7.6%). In contrast, the number of fluorographic mobile units declined slightly from 170 to 159 (−6.5%), possibly due to a strategic shift toward stationary or centralized imaging facilities.

### 3.2. Diagnostic Radiology Human Resources

Over the study period, the diagnostic radiology workforce expanded notably (Table 1). Radiology doctors increased from 3529 in 2018 to 4511 in 2024 (+27.8%). Similarly, the number of radiologists rose from 1567 to 1909 (+21.8%). The number of ultrasound doctors increased substantially from 1396 in 2018 to 2178 in 2024 (+56.1%), indicating growing reliance on non-ionizing imaging modalities. Radioisotope doctors, although a small group, increased from 9 to 32 (+255.6%). X-ray technicians also grew from 3490 to 4244 (+21.6%), supporting the operational expansion of imaging services.

### 3.3. Outpatient Radiology Consultations

Outpatient visits to radiologists exhibited fluctuating trends (Table 1). After an initial decline from 757,282 in 2018 to 488,541 in 2020 (−35.5%), likely due to the COVID-19 pandemic, visits peaked at 980,223 in 2022 before falling again to 479,325 in 2024. A similar pattern was observed for outpatient visits for medical conditions: they declined from 703,877 in 2018 to 355,471 in 2020, then increased to 482,488 in 2022, and then slightly decreased to 404,885 in 2024. These fluctuations may reflect pandemic-related service disruptions, followed by post-pandemic recovery and subsequent stabilization.

### 3.4. Diagnostic Imaging Procedure Volumes

The volume of diagnostic imaging procedures also shifted notably (Table 1). X-ray examinations declined significantly from 20.6 million in 2018 to 11.2 million in 2020, then rose gradually to 14.6 million in 2024, resulting in an overall decrease of approximately 29.2% over the period. Fluorography procedures experienced an even more pronounced drop, from 28.3 million in 2018 to 9.1 million in 2024, representing a 67.7% decrease, likely indicating a reduction in population-based screening or a transition to more targeted diagnostic approaches. In contrast, CT usage rose steadily from 491,738 in 2018 to 1.6 million in 2024, marking an impressive increase of over 220%, consistent with global trends favoring high-resolution imaging in clinical diagnostics.

**Figure 1:**
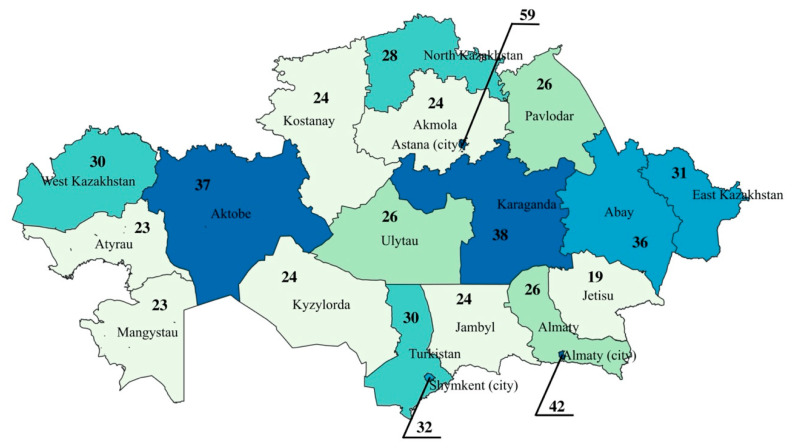
Availability of Radiology Physicians per 100,000 Population by Region in Kazakhstan in 2024.

Figure 1 illustrates the availability of radiology physicians per 100,000 population across Kazakhstan’s regions in 2024. The highest concentration of radiologists is in Astana, where 59 radiologists per 100,000 population are reported. This is considerably higher than in other regions and likely reflects the centralized nature of healthcare services, infrastructure, and workforce in the capital. Almaty and Shymkent also show relatively high availability with 42 and 32 radiologists per 100,000, respectively, highlighting a broader trend of higher healthcare workforce density in major urban centers.

In contrast, less developed regions have lower levels of radiologist availability. Jetisu reports the lowest figure at 19 per 100,000, followed closely by Atyrau and Mangystau, each with 23. These numbers point to a potentially critical shortage of diagnostic capacity in several parts of the country. Mid-range values are found in regions such as Karaganda (38 per 100,000), Aktobe (37 per 100,000), and Abay (36 per 100,000), which exceed the national average and may serve as regional hubs for medical services. A cluster of regions, including Akmola, Kostanay, Kyzylorda, and Jambyl, consistently reports around 24 radiologists per 100,000, indicating moderate but potentially suboptimal availability.

**Figure 2:**
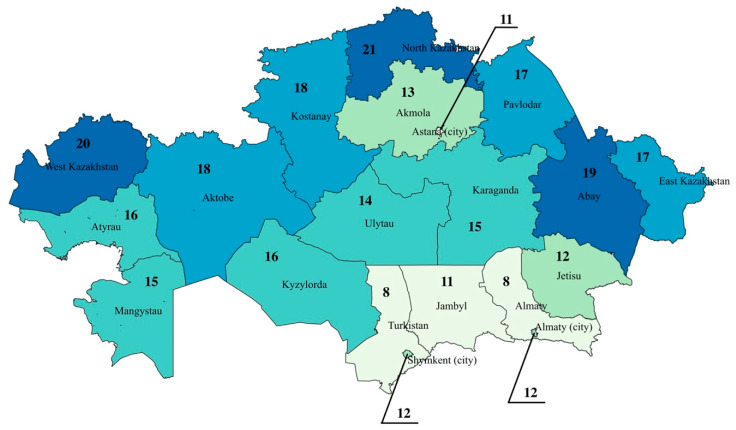
Regional Distribution of Diagnostic Radiology Equipment per 100,000 Population in Kazakhstan in 2024.

The regional distribution of diagnostic radiology equipment per 100,000 population in Kazakhstan in 2024 reveals significant disparities across the country (Figure 2). The highest availability is observed in North Kazakhstan with 21 units per 100,000 population, followed by West Kazakhstan (20 per 100,000) and Abay Region (19 per 100,000). These regions stand out as relatively well-equipped, potentially reflecting better healthcare infrastructure or targeted regional investments in diagnostic technology.

In contrast, the lowest levels of equipment availability are concentrated in the southern regions. Turkistan and Almaty region report only 8 units per 100,000, with Jambyl and Astana slightly higher at 11 per 100,000, and Almaty (city) and Jetisu at 12 per 100,000.

Table 2 presents demographic and occupational characteristics of 177 medical workers stratified into three professional groups: Diagnostic Radiology Personnel (57%), Interventional Physicians (25%), and Interventional Support Staff (18%). The mean age across all participants was 41 years (±11), with no significant difference between groups (*p* = 0.435). Gender distribution showed a predominance of females (51%), with higher proportions of women among the Interventional Support Staff (63%) and Diagnostic Radiology Personnel (54%) than among Interventional Physicians (38%). However, this difference did not reach statistical significance (*p* = 0.075).

The majority of participants were employed in diagnostic healthcare facilities (72%), with a similar distribution across all occupational groups (*p* = 0.437).

Importantly, the mean quarterly individual radiation dose was significantly different among the groups (*p* = 0.001). Interventional Physicians had the highest average dose (0.65 ± 0.58 mSv), followed by Diagnostic Radiology Personnel (0.44 ± 0.20 mSv), and Interventional Support Staff (0.43 ± 0.14 mSv).

A cross-sectional survey of 324 radiology specialists in Kazakhstan examined anxiety related to occupational exposure to ionizing radiation and its association with socio-demographic and professional factors (Table 3). Overall, 32% of respondents reported anxiety, while 68% did not. Age was significantly associated with anxiety (*p* < 0.001), with higher concern among mid-career professionals (36–55 years). Gender (*p* = 0.216) and education level (*p* = 0.201) showed no significant association.

Job type was strongly linked to anxiety (*p* < 0.001). Radiologists working with ionizing radiation had lower anxiety levels. At the same time, those using non-ionizing techniques and X-ray technicians were more frequently anxious, possibly due to perceived vulnerability and less control over exposure (Table 3). Residence also influenced anxiety (*p* = 0.001), with rural professionals more likely to report concerns than their urban counterparts. Work experience was not significantly associated with anxiety (*p* = 0.489).

As shown in Table 4, the majority of participants worked 31–40 h per week (43%), followed by 41+ h (28%), and workload was not significantly associated with radiation-related anxiety (*p* = 0.646). However, significant associations were found between protective practices and anxiety. For instance, 58% of those who expressed anxiety reported not wearing protective aprons, compared to 27% of those without anxiety (*p* < 0.001). Dosimeter use also showed a significant association: 27% of anxious workers did not use dosimeters, compared with only 12% of non-anxious respondents (*p* = 0.001).

Training and education on radiation safety appeared to influence anxiety levels. Among those reporting anxiety, 27% believed they had inadequate training, compared to only 6% in the non-anxious group (*p* < 0.001). Job satisfaction differed significantly between groups; 35% of anxious respondents were dissatisfied with their work versus 17% of those without anxiety (*p* < 0.001). Dissatisfaction with working conditions was primarily linked to low wages (58%) and excessive workload (41%), with anxious individuals more likely to cite low wages (75% vs. 50%, *p* < 0.001) and insufficient radiation protection (25% vs. 6%, *p* < 0.001). Although work-related health problems were more often reported by anxious respondents (15% vs. 9%), this difference was not statistically significant (*p* = 0.092). Furthermore, higher levels of both situational (58%) and personal anxiety (56%) were more prevalent among those worried about radiation exposure (*p* < 0.001 and *p* = 0.002, respectively).

## 4. Discussion

This study provides a comprehensive assessment of diagnostic radiology services and the occupational health status of radiology personnel in Kazakhstan. Our findings demonstrate that while diagnostic imaging infrastructure and human resources have steadily expanded over the past six years, disparities in regional availability and variations in occupational radiation exposure and related anxiety remain pronounced. Notably, the expansion of advanced imaging modalities, such as computed tomography, has outpaced the growth of conventional radiographic procedures, reflecting a shift in diagnostic strategies. Although data on individual radiation exposure were available only from facilities in the capital, they revealed that considerable proportions of radiology professionals—particularly those with limited access to radiation protection measures or training—reported elevated anxiety related to ionizing radiation exposure.

The observed regional disparities in the availability of radiologists and diagnostic equipment mirror global patterns, in which urban centers tend to attract more qualified personnel and investment in infrastructure [19]. Similar inequalities have been documented in Brazilian [20], US [21], and Indian [22] healthcare systems, suggesting systemic challenges in decentralizing high-quality diagnostic services. The regional distribution of diagnostic radiology equipment shows a marked shortage in the southern part of the country, including large urban centers such as Almaty. This is particularly notable given the comparatively high concentration of radiology physicians in these areas. The mismatch suggests that although human resources tend to cluster in major cities, the expansion of diagnostic infrastructure has not kept pace, potentially constraining service capacity despite adequate specialist availability.

Our study revealed a marked decline in the number of fluorographic and conventional X-ray examinations, alongside a substantial increase in computed tomography utilization. This trend may reflect a growing clinical preference for more advanced imaging modalities, such as CT, which, despite their higher radiation dose and cost, provide greater diagnostic precision. The increase in CT use has been documented in other countries, particularly in the post-COVID-19 era, when the pandemic underscored the value of high-resolution imaging for rapid and accurate disease assessment [23,24]. The significant growth in CT use in Kazakhstan aligns with international trends that emphasize the diagnostic value of cross-sectional imaging [25], yet it also underscores the need to monitor radiation safety more closely. A meta-analysis involving 111.6 million adults across Asia, Europe, and the Americas revealed a significantly elevated risk of cancer associated with CT scan exposure in adults [26], raising public health considerations as imaging volumes increase.

The findings of this study indicate a greater radiation exposure burden among physicians performing interventional procedures, likely due to the complexity and duration of fluoroscopy-guided interventions. This is consistent with findings from multicenter studies in the US [27] and Europe [28]. This increased exposure is likely due to the procedural complexity and real-time imaging demands inherent in interventional work. Furthermore, our survey of radiology professionals revealed that multiple factors, including age, job type, protective behavior, and training adequacy, influence anxiety related to radiation exposure. These findings align with earlier reports indicating that insufficient education in radiation safety correlates with higher levels of occupational stress and risk perception [29,30]. Interestingly, personnel using non-ionizing modalities or working in rural settings reported greater anxiety, potentially reflecting limited institutional support and access to updated protective measures.

The association between poor protective practices, such as failure to wear aprons or use a dosimeter, and increased anxiety is particularly concerning. This suggests that anxiety may not only reflect perceived health risks but also stem from a lack of empowerment or resources to mitigate those risks. Mandatory radiation protection training is required every five years for all radiology personnel, including physicians, technicians, and staff working with ionizing and non-ionizing modalities. Training for overexposed workers includes additional on-site instruction as required by national regulations. The findings of this study parallel evidence from a systematic review of 12 articles from different countries, which showed that improved access to personal protective equipment and ongoing education significantly reduce stress among healthcare workers exposed to radiation [31]. Job dissatisfaction, driven primarily by low wages and high workloads, further compounds occupational stress and may affect performance and retention in the radiology workforce. These factors likely contribute not only to general job-related stress but also to heightened anxiety about radiation exposure, particularly among those in operational roles such as X-ray technicians. The combination of perceived health risks, insufficient compensation, and overwhelming workloads may create a sense of vulnerability and burnout, ultimately impacting both mental well-being and the quality of care provided [32,33]. Although a direct causal link between radiation exposure and psychological distress is difficult to establish, our results highlight the interplay between occupational health, safety culture, and mental well-being.

Among the strengths of this study is the integration of national administrative data with primary survey findings, allowing for a holistic evaluation of both system-level trends and individual-level experiences. Our stratified analysis by job type, setting, and protective behaviors offers actionable insights for targeted interventions. However, several limitations should be noted. First, the cross-sectional design precludes causal inference. Second, self-reported measures of anxiety and protective practices may be subject to recall or social desirability bias. Third, using quarterly averages for radiation exposure limits temporal resolution and may fail to capture short-term spikes during high-intensity or emergency procedures, potentially underestimating actual risk levels.

## 5. Conclusions

The radiology workforce in Kazakhstan faces increasing demands and uneven resource distribution, with significant occupational radiation exposure among interventional professionals. A substantial subset of workers report anxiety linked to inadequate training and insufficient protective measures. National strategies should prioritize equitable distribution of diagnostic infrastructure, regular radiation safety training, and improved working conditions to safeguard both the physical and mental health of radiology personnel.

## Figures and Tables

**Table 1:** Summary of Diagnostic Radiology Infrastructure, Personnel, Service Utilization, and Procedures in Kazakhstan (2018–2024).

	Year			
	2018	2020	2022	2024
Diagnostic Imaging Facilities				
Radiology rooms	1127	1134	1227	1285
CT rooms	162	200	282	358
Fluorography rooms	815	789	835	877
Fluorographic mobile units	170	150	164	159
Diagnostic Radiology Staff				
Radiology doctors	3529	3773	4047	4511
Radiologists	1567	1574	1702	1909
Ultrasound doctors	1396	2032	2138	2178
Radioisotope doctors	9	15	34	32
X-ray technicians	3490	3494	3844	4244
Outpatient Radiology Consultations				
Outpatient visit to a radiologist	757,282	488,541	980,223	479,325
Outpatient visit to a radiologist for a medical condition	703,877	355,471	482,488	404,885
Diagnostic Imaging Procedures				
X-ray examinations	20,627,600	11,224,050	12,837,500	14,620,649
Fluorography	28,273,440	9,480,051	10,621,800	9,120,138
CT	491,738	810,479	1,321,430	1,575,068

**Table 2:** Characteristics of Medical Personnel by Occupational Group and Quarterly Radiation Dose Exposure (*n* = 177).

	Total (*n* = 177)	Diagnostic Radiology Personnel (*n* = 100, 57%)	Interventional Physicians (*n* = 45, 25%)	Interventional Support Staff (*n* = 32, 18%)	*p*-Value
Age (years), μ±SD	41 (11)	43 (12)	39 (9)	40 (12)	0.435
Gender, *n* (%)					0.075
Female	91 (51)	54 (54)	17 (38)	20 (63)	
Male	86 (49)	46 (46)	28 (62)	12 (37)	
Type of healthcare facility, *n* (%)					0.437
Diagnostic	127 (72)	74 (74)	33 (73)	20 (63)	
Emergency	50 (28)	26 (26)	12 (27)	12 (37)	
Quarterly Individual Radiation Dose (mSv), μ±SD	0.49 (0.34)	0.44 (0.20)	0.65 (0.58)	0.43 (0.14)	0.001

**Table 3:** Socio-demigraphic data of survey respondents (*n* = 324).

	Total (*n*= 324)	Are You Worried or Anxious About Being Exposed to Ionizing Radiation at Work?		
		No (*n* = 220, 68%)	Yes (*n* = 104, 32%)	*p*-Value
Age, *n* (%)				<0.001
19–25 y.o.	10 (3)	6 (3)	4 (4)	
26–35 y.o.	108 (34)	88 (40)	20 (19)	
36–45 y.o.	92 (28)	46 (21)	46 (44)	
46–55 y.o.	92 (28)	60 (27)	32 (31)	
56–63 y.o.	22 (7)	20 (9)	2 (2)	
Gender, *n* (%)				0.216
Female	196 (60)	128 (58)	68 (65)	
Male	128 (40)	92 (42)	36 (35)	
Job, *n* (%)				<0.001
Radiologists working with ionizing radiation	174 (54)	126 (57)	48 (46)	
Radiologists working with non-ionizing radiation	44 (13)	18 (8)	26 (25)	
X-ray technicians	106 (33)	76 (35)	30 (29)	
Education, *n* (%)				0.201
Bachelor’s degree	166 (51)	110 (50)	56 (4)	
Residency	130 (40)	86 (39)	44 (42)	
Master’s degree	18 (6)	16 (7)	2 (2)	
Ph.D. degree	10 (3)	8 (4)	2 (2)	
Living area, *n* (%)				0.001
Rural	22 (7)	8 (4)	14 (13)	
Urban	302 (93)	212 (96)	90 (87)	
Experience, *n* (%)				0.489
0–5 years	96 (30)	70 (32)	26 (25)	
6–10 years	60 (19)	36 (16)	24 (23)	
11–15 years	74 (23)	48 (22)	26 (25)	
16–20 years	34 (10)	22 (10)	12 (11)	
21–25 years	34 (10)	26 (12)	8 (8)	
26 years and more	26 (8)	18 (8)	8 (8)	

**Table 4:** Comparison of occupational and psychological factors among radiology specialists with and without anxiety related to ionizing radiation exposure in Kazakhstan.

	Total	Are You Worried or Anxious About Being Exposed to Ionizing Radiation at Work?		
		No (*n* = 220, 68%)	Yes (*n* = 104, 32%)	*p*-Value
Working environment satisfaction				
Workload per week, *n* (%)				0.646
less than 15 h	18 (6)	14 (6)	4 (4)	
15–30 h	76 (23)	48 (22)	28 (27)	
31–40 h	140 (43)	96 (44)	44 (42)	
more than 41 h	90 (28)	62 (28)	28 (27)	
Do you wear a protective apron at work?				<0.001
No	120 (37)	60 (27)	60 (58)	
Yes	92 (28)	66 (30)	26 (25)	
Unnecessary	112 (35)	94 (43)	18 (17)	
Do you wear dosimeters?, *n* (%)				0.001
No	54 (17)	26 (12)	28 (27)	
Yes	270 (83)	194 (88)	76 (73)	
“I feel that you have adequate training and education regarding radiation safety issues”, *n* (%)				<0.001
No	42 (13)	14 (6)	28 (27)	
Perhaps	114 (35)	84 (38)	30 (29)	
True	98 (30)	66 (30)	32 (31)	
Absolutely right	70 (22)	56 (26)	14 (13)	
“I am satisfied with my work”, *n* (%)				<0.001
No	74 (23)	38 (17)	36 (35)	
Yes	178 (55)	142 (65)	36 (34)	
Difficult to answer	72 (22)	40 (18)	32 (31)	
Reason for dissatisfaction with working conditions, *n* (%)				
Fully satisfied	88 (27)	80 (36)	8 (8)	<0.001
Low wages	188 (58)	110 (50)	78 (75)	<0.001
Excessive workload	132 (41)	82 (37)	50 (48)	0.065
Low protection from ionizing radiation	40 (12)	14 (6)	26 (25)	<0.001
Health problems caused by work	36 (11)	20 (9)	16 (15)	0.092
Spielberger’s anxiety scales				
Situational anxiety, *n* (%)				<0.001
Low	196 (60)	152 (69)	44 (42)	
High	128 (40)	68 (31)	60 (58)	
Personal anxiety, *n* (%)				0.002
Low	184 (57)	138 (63)	46 (44)	
High	140 (43)	82 (37)	58 (56)	

## Data Availability

Data are available from the corresponding author, Galiya Orazova, upon reasonable request and with permission of the Salidat Kairbekova National Research Center for Health Development.

## Supplementary Materials

The following supporting information can be downloaded at: https://www.mdpi.com/article/10.3390/ijerph22121785/s1, Table S1: Survey for radiologists and X-ray technicians working in the Republic of Kazakhstan.

## Abbreviations

CT	computed tomography
COVID-19	coronavirus disease 2019
IR	ionizing radiation
mSv	millisievert
NCJSC	Non-Commercial Joint-Stock Company
STAI	State-Trait Anxiety Inventory
US	ultrasound
