# Supplementary material for: Diagnostic Radiology Services and Occupational Radiation Anxiety in Kazakhstan
Source: Int J Environ Res Public Health. 2025 Nov 25;22(12):1785. doi: 10.3390/ijerph22121785 (PMC12732935; doi:10.3390/ijerph22121785)
Supplement: Supplementary file 1 [file ijerph-22-01785-s001.zip › ijerph-3986162-supplementary.pdf]

Table S1: Survey for radiologists and X-ray technicians working in the Republic of Kazakhstan

Dear respondent!

This study is aimed at identifying the psychological stress and anxiety associated with the work of radiologists and X-ray technicians in the Republic of Kazakhstan. The survey results will be used to develop recommendations for disease prevention among radiologists and X-ray technicians and to improve the quality of their working conditions in the country.

You will need to answer a number of questions related to your daily life. Please answer honestly and do not skip questions.

The information received about the respondents is confidential and is not subject to disclosure. The information obtained as a result of the survey will be used for scientific purposes.

By continuing the survey, you voluntarily agree to take part in the study.

1. Your profession at the time of the interview
    - o Radiologist
    - o CT scanner
    - o MRI doctor
    - o Ultrasound
    - o Multimodal radiologist
    - o Interventional radiologist
    - o X-ray laboratory assistant (X-ray room)
    - o X-ray laboratory assistant (CT room)
    - o X-ray laboratory assistant (MRI room)
    - o X-ray laboratory assistant (mammography room)
    - o X-ray laboratory assistant (fluorography room)
    - o X-ray laboratory assistant (interventional radiology)

2. Your gender:
    - o Man
    - o Woman

3. Your age:
    - o 19-25 years old
    - o 26-35 years old
    - o 36-45 years old
    - o 46-55 years old
    - o 56-63 years old
    - o 63 years and more

4. How many years have you been working in radiology diagnostics?

- 0-5 years
- 6-10 years
- 11-15 years
- 16-20 years
- 21-25 years
- 26 or more

5. Region of the medical organization in which you work
   - Abay region
   - Akmola region
   - Aktobe region
   - Almaty region
   - Atyrau region
   - East Kazakhstan region
   - Zhambyl region
   - Zhetysu region
   - West Kazakhstan region
   - Karaganda region
   - Kostanay region
   - Kyzylorda region
   - Mangystau region
   - Pavlodar region
   - North Kazakhstan region
   - Turkestan region
   - Ulytau region
   - Astana city
   - Almaty city
   - Shymkent city

6. The level of the medical organization in which you work
   - Rural
   - Urban

7. Level of education
   - Bachelor’s degree
   - Residency
   - Master’s degree
   - PhD

8. How many hours a week do you work?
   - up to 15 hours
   - up to 30 hours
   - up to 40 hours
   - more than 41 hours

9. Do you wear a protective apron at work?
   - Yes
   - No
   - No need

10. Do you feel that you have sufficient preparation and training on radiation safety issues
    - No, this is not so
    - Perhaps so
    - Yes
    - Absolutely right

11. Do you wear dosimeters and what kind?
    - Only on-body dosimeter
    - Under the apron, for the eyes
    - Under the apron, for the eyes, for the hands
    - Under the apron, for hands
    - No

12. Are you satisfied with your job?
    - Satisfied
    - Not satisfied
    - I find it difficult to answer

13. What is the reason for dissatisfaction with working conditions?
    - You can choose several answers
    - I am happy with everything
    - Low salary
    - Excessive workload
    - Poor protection from ionizing radiation
    - Health problems caused by work

14. Do you feel anxious or worried about exposure to ionizing radiation at work?
    - No
    - Yes
    - Sometimes
